# Deaths of Medical Men

**Published:** 1859-04

**Authors:** 


*Deaths of Medical Men.*—We see by the Savannah Journal of Medicine, that Dr. George M. Newton, Emeritus Prof. of Anatomy in the Medical College of Georgia, has lately died. He was a man of eminent ability and a fine lecturer.

Peter C. Gaillard, M. D., Professor of the Theory and Practice of Medicine in the Medical College of South Carolina, departed this life in Charleston, on the 14th day of January last, at the comparatively early age of forty-four.—*Savannah Journal of Medicine.*

*Prof. Thos D. Mütter.*—We see by the daily papers, that this eminent surgeon lately died at Charleston, S. C. Dr. Mütter was long the Prof. of Surgery in the Jefferson Medical College of Philadelphia, and was one of the most popular lecturers on this branch that we have ever had. Ill health, however, compelled him a few years ago, to retire from teaching; when his place was filled by Dr. Gross.

Also, Dr. J. C. Goble, of Newark, N. J., formerly President of the State Medical Society.

Prof. Ellet, of South Carolina University.

Dr. Edward Hudson, Surgeon, U. S. Navy.

Died, in the city of Alexandria, Egypt, after a short illness, on the 23d of January last, Doctor George Abbott, American Vice-Consul, late of this Auburn, N. Y.

The remains of the deceased were followed to their final resting place by the American consul at Alexandria, the officers and crew of the U. S. sloop of war Macedonia, the bier being drawn by twelve seamen from the sloop, and enveloped with the American flag.

Dr. Abbott spent his boyhood in this city, pursued his medical studies in the office of Dr. Briggs, and graduated from the University of Buffalo.

(Dr. Abbott is well remembered by the faculty of the Buffalo Medical College. He was a fine scholar and a complete gentleman. He was the son of the late Mr. Abbott, of Auburn, and brother of Dr. Abbott, of Cairo, who made the famous Egyptian collection now in New York.)

